# Systematic Review of Sleep Duration and Development of Myopia

**DOI:** 10.7759/cureus.56216

**Published:** 2024-03-15

**Authors:** Omna Chawla, Anupam Singh, Devesh Kumawat, Nilotpal Chowdhury, Barun Kumar

**Affiliations:** 1 Department of Physiology, Government Doon Medical College, Dehradun, IND; 2 Department of Ophthalmology, All India Institute of Medical Sciences, Rishikesh, Rishikesh, IND; 3 Department of Ophthalmology, All India Institute of Medical Sciences, New Delhi, New Delhi, IND; 4 Department of Pathology and Laboratory Medicine, All India Institute of Medical Sciences, Rishikesh, Rishikesh, IND; 5 Department of Cardiology, All India Institute of Medical Sciences, Rishikesh, Rishikesh, IND

**Keywords:** systematic review, myopia progression, myopia, sleep duration, sleep

## Abstract

There is a knowledge gap in the relationship between sleep duration and myopia. Since sleep duration is a modifiable risk factor, its association with the development and progression of myopia has implications for public health. This review was conducted in accordance with the 2020 Preferred Reporting Items for Systematic Reviews and Meta-Analyses (PRISMA) guidelines. The bibliographic databases of PubMed and Scopus were searched for published studies on the association between sleep duration and myopia. These databases were searched in December 2023 with no date or study design limits. The relevant literature was extracted and met the priori determined population (children, adolescents, and adults suffering from myopia with or without corrective glasses), intervention/exposure (sleep), and the outcome (various indicators of sleep especially sleep duration/bedtime/wake time and sleep quality). Data were gathered by gender, age, and refraction technique and standardized to the definition of myopia as refractive error ≥0.50 diopter. The relevant literature was extracted from these electronic databases using the keywords "sleep," "sleep duration," "bedtime," and "myopia." English language articles related to the topic were included. Articles that have discussed the role of risk factors for myopia but did not mention any relation to sleep were excluded. Sixteen studies were included after reviewing the relevant literature, and only six studies have shown a significant relationship between shorter duration of sleep and the development of myopia. This review suggests that apart from other environmental factors, sleep duration may have a role in developing myopia. Thus, increasing awareness about optimum sleep duration has a potential utility to reduce the development and progression of myopia.

## Introduction and background

Myopia is an error of refraction due to an increase in the eyeball's axial length. It leads to the blurring of the image as the light rays focus in front of the retina. Approximately 4,758 million people worldwide will be myopic by 2050 [1]. These figures imply an alarming picture that half of the world's population will be myopic by 2050. It is a challenge for the public health system as myopia management involves supplying the appropriate corrections for the refractive error and dealing with long-term pathological complications, such as retinal detachment myopic degenerations, choroidal neovascularization, and the risk of glaucoma. Moreover, these complications cannot be prevented by just the correction of refractive error [2].

Various studies have identified genetic susceptibility loci for myopia; however, some studies have suggested that gene-environment interaction strongly influences the risk of myopia [3-5]. The genetic-environmental interaction index indicated that genetic factors contribute only 12.5% compared to environmental factors in the formation of myopia [6]. Recent reports have shown a significant negative association with outdoor time, physical activity, and good nutrition [7,8]. Even the increased outdoor time prevents the onset of myopia and slows its progression [9]. Research focusing on natural light exposure and myopia risk suggests that encouraging children to spend more time outdoors, even during dim light conditions, may help to reduce the incidence and progression of myopia [10]. Similarly, the appropriate spectral composition and intensity of light and the timing of light exposure are critical in the proper development of the eye and refraction [11]. It is worth mentioning that there exists a network of circadian clocks within the retina and in the eye, which synchronizes the day/night cycle [12,13]. Moreover, the axial length and choroidal thickness exhibit cyclical variations over the day and affect eye growth and development of myopia [14,15].

There is a prevailing hypothesis that the alteration of circadian rhythms plays a role in myopia development. Some have suggested that disrupted sleep is associated with the development of myopia [16]. However, the association between myopia and sleep duration has not received the needed attention. Surprisingly, insufficient sleep across the globe is also emerging as a parallel epidemic with myopia [17]. However, the evidence is unclear that the two may be interrelated. Thus, this manuscript aims to provide an updated review of the literature assessing the relationship between sleep and myopia.

## Review

The strategy of systematic searching was adopted using the electronic databases of PubMed and Scopus. The review analyzed children, adolescents, and adults without known sleep disorders but suffering from myopia with or without corrective glasses. The Participants, Interventions, Comparisons, Outcomes, and Study Design (PICOS) framework was followed to identify key study concepts in the research question and to facilitate the search process (Table 1). 

**Table 1:** Selection criterion for the included studies

Criterion	Inclusion
Research question	Clear mention about the association between sleep duration/sleep parameters and myopia prevalence/progression
Type of study	Cross-sectional, cohort, case-control, intervention trials, or retrospective studies
Age of study participants	All age groups
Language	English
Same study	Only one article; the one with a clear statement about sleep duration /bedtime /wake time/sleep quality
The outcome in terms of myopia	Self-reported myopia/use of glasses using a questionnaire or refraction done with or without cycloplegia
Sleep parameters	Clear reporting of sleep duration in terms of hours per day or bedtime or wakeup time

The population of interest was children, adolescents, and adults who were suffering from myopic error of refraction. We tried to keep the focus on the general myopic population and not specific clinical populations. Sleep duration is taken as the intervention or exposure for this review. Sleep duration could be reported in many ways and could include total sleep duration (i.e., per 24-hour period, including naps, or nighttime sleep duration only).

Studies were eligible if they measured sleep using polysomnography or actigraphy or subjective assessment using a questionnaire-based, self-report, or proxy report. The methods to report myopia include cycloplegic or non-cycloplegic refraction or self-reporting of the use of glasses. The outcome included various indicators of sleep, especially sleep duration/bedtime/wake time and sleep quality. Various levels of sleep duration were used for comparison. However, a comparator or control group was not required for inclusion. We excluded studies that had not reported the duration of sleep. Keywords used in various combinations were "sleep," "sleep duration," "sleep quality," and "myopia." Titles and abstracts of potentially relevant articles were screened. The articles were included based on relevance to the research question. The published peer-reviewed original English language manuscripts were eligible for inclusion. The data were extracted and documented using a predesigned Performa. The information was gathered on authors, year of publication, country of study, study design, study population characteristics like age and sample size, method of myopia detection, sleep-related parameters, and significance of the association. The disparities in population characteristics, study methodologies, and measurements employed to assess sleep and myopia prevent their quantitative merging in a meta-analysis. Consequently, only a qualitative synthesis was undertaken to summarize the findings.

Results

**Figure 1:**
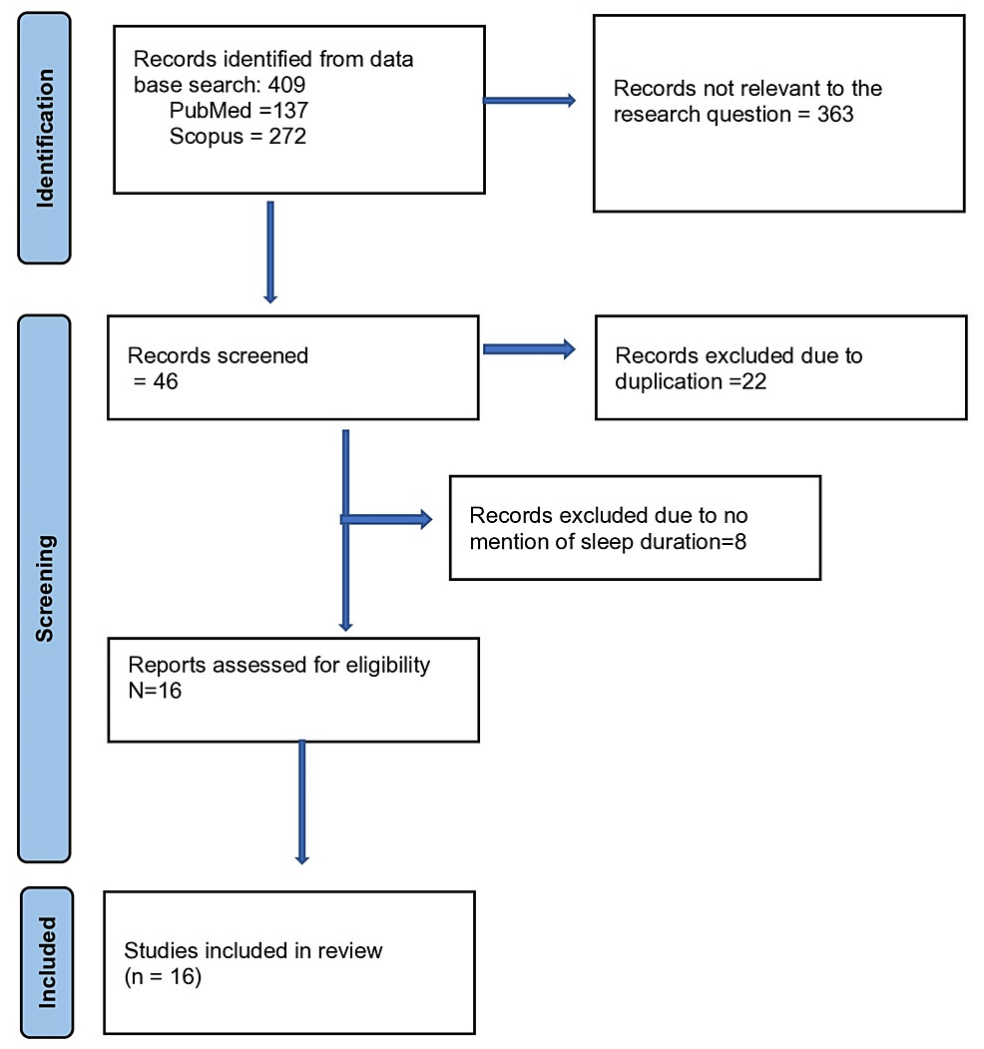
PRISMA flow chart showing the process of selecting articles/studies for the systematic review. PRISMA: Preferred Reporting Items for Systematic Reviews and Meta-Analyses

The search strategy identified 409 articles from the electronic search databases (Figure 1).

The search included the articles updated till 31st of December 2023. The abstracts of those 409 studies were studied independently by all authors. A total of 363 abstracts were not relevant to the research question. Furthermore, 22 abstracts were excluded because of duplication, and eight were excluded due to no mention of sleep duration. Finally, 16 abstracts were found relevant as per the inclusion criterion. The manuscripts of all 16 studies were obtained.

Sleep assessment

All 16 studies have reported on sleep using questionnaires. However, only two [18,19] have used a specific sleep questionnaire, such as the Pittsburgh Sleep Quality Assessment (PSQI) to measure the quality and patterns of sleep, which is a validated instrument [20]. One study has used actigraphy, which estimates sleep-wake patterns using a wrist-worn monitor. Actigraphy is also a validated method to assess sleep parameters and highly correlates with polysomnography [21]. Polysomnography is the gold standard for recording sleep and related parameters of sleep objectively [22]. We could not find any study that used polysomnography to assess sleep parameters in relation to myopia. In the present review, we have focused on sleep duration, usually reported in hours per day. One study has reported total sleep duration in hours per week.

Myopia assessment

In most of these studies, the refractive error was measured by refraction with or without cycloplegia. Myopia and high myopia were defined as ≤-0.50 dioptres (D) and ≤-6.0 D, respectively. There is a lack of uniformity in the methods and criteria for determining persons as myopic, and thus it is difficult to compare the results of different studies. However, all studies have defined the standard used for documentation of refractive error. Other methods to document myopia in these studies were self-reports or proxy by a parent or guardian. The self-reporting of myopia is also sensitive and specific (0.76 and 0.74, respectively) for identification [23].

Observation

Studies in different populations report that those with myopia were more likely to have a shorter sleep duration than those without myopia. A survey of Korean schoolchildren found that the prevalence of myopia was high (87.7%) in those who slept for less than five hours compared to those who slept for more than nine hours (78.1%). The odds of getting myopia were 41% less for those sleeping more than nine hours than those with less than five hours of sleep [24].

Similarly, a study conducted on Chinese schoolchildren found that 68.45 % of myopic children slept for less than seven hours [25]. In a cross-sectional study of Japanese children, myopes slept one hour less than non-myopes [19]. The university students in Australia who were myopes had a sleep duration of only 7.18 hours compared with 8.46 hours in emmetropes [18]. The data from the Chinese National Survey on Students' Constitute and Health (CNSSH) found that sleep duration was less than seven hours in post-menarche girls with myopia compared to nine hours or more in emmetropes [26]. The studies also revealed that short sleep duration as a risk of myopia was a relatively independent factor when adjusted for other confounders [24,25]. The change in the odds for myopia was 0.03 (crude odds ratio (OR) of 0.87 to an adjusted OR of 0.90) when adjusted for other confounders, like sex, age, education levels, economic status, height, and physical activity.

Similarly, Gong et al. found that children who slept for less than seven hours per night had a 3.37 times higher risk of myopia than those with greater than nine hours of sleep [25]. Moreover, Jee et al. showed a dose-response pattern between sleep duration and myopia. They reported that one one-hour increase in sleep reduced the risk of myopia by 10%. Even the absolute refractive error decreased by 0.1 D with an increased sleep duration of one hour. In line with these findings, a study on parents' attitudes found that the parents who ensured sufficient sleep for their children reduced the risk of myopia in school-age children [27].

The ROAM (Role of Outdoor Activity in Myopia) study found that 53% of myopes in the age group of 10-15 years slept less than recommended hours. Still, there was no significant difference in sleep duration between myopes and non-myopes [28]. However, sleep latency was low for myopes by approximately 3.8 minutes compared to non-myopes. Furthermore, they found that myopes had more variable sleep duration across days and seasons than non-myopic children [28]. A Shanghai study in a prospective trial of 24 months found no relationship between sleep duration and myopia. However, they suggested that children with later bedtimes had a higher risk for myopia onset [29]. Similarly, a retrospective cross-sectional study of 1831 suburban children aged 11-18 years using a questionnaire in Qingpu, China, found no statistically significant association between sleep duration and myopia. However, they also found that sleep habits like bedtime and wake time are significantly associated with myopia [30]. A clinical trial involving urban primary school Chinese children of mean age 9.8 years +/-0.44 found no statistically significant association between total sleep duration (midday and nighttime) and myopia [31]. However, the sleep habit questionnaire scores and bedtime resistance scores were significantly abnormal in myopes. Sleep duration (in hours per week) did not correlate with the onset of myopia in a three-year prospective cohort of 522 boys [32]. The questionnaire-based study in Nanjing University students found a high prevalence of myopia (86.8%). Still, there was no significant difference between sleep duration among myopes and non-myopes [33]. A study following a birth cohort found that 3.5% of participants were myopic at three years of age. However, sleep duration and number of night awakenings at 12 months of age had no significant association with myopia development [34].

Some studies found that sleep latency in myopes was higher as compared to emmetropes. The Japanese myopic children went to bed 74 minutes later than the non-myopes [19]. An actigraphy-based study also reported a delay in sleep onset by 56 minutes among myopes compared to emmetropes [18].

The Anyang childhood eye study observed a cohort of children aged five to nine years for four years for myopia progression, and axial elongation reported no significant association between sleep duration and axial elongation [35]. A brief description of all the studies included in this systematic review is presented in Table 2.

**Table 2:** List of included studies with a brief description of characteristic findings.

Serial No.	Study	Title of the study	Study design/ country	No. of participants	Age of study population	Method used to measure sleep	Method used to record myopia	Significant finding
1.	Chakraborty et al., 2021 [18]	Myopia, or near-sightedness, is associated with delayed melatonin circadian timing and lower melatonin output in young adult humans	Observational study/ Australia	32	18 to 25 years (22.06+/-2.35)	The Pittsburgh Sleep Quality Index (PSQI) and the Morningness-Eveningness Questionnaire and Actigraphy	Non-cycloplegic autorefraction	Myopes had shorter sleep duration. greater sleep onset latency than emmetropes.
2.	Xu et al, .2020 [26]	The association between menarche and myopia and its interaction with related risk behaviors among Chinese school-aged girls: a nationwide cross-sectional study	National survey/ Mainland China	102,883 girls	10-15 years	Questionnaire	Non-cycloplegic refraction	Sleep duration of less than seven hours increased the risk for myopia in post-menarche.
3.	Ostrin et al., 2020 [28]	Sleep in myopic and non-myopic children	Prospective longitudinal examination over multiple visits Brisbane/Australia	91	10-15 years (13.02+/-1.37)	Wrist-worn actigraphic device (actiwatch)	Non-cycloplegic refraction	Myopic children had more variable sleep duration than non-myopes. Sleep latency is shorter in myopes (suggestive of sleep debt).
4.	Liu et al., 2020 [29]	Sleeping late is a risk factor for myopia development amongst school‑aged children in China	Two-year prospective clinical trial Shanghai/China	409	6 to 9 years	Questionnaire via a mobile phone app	Cycloplegic autorefraction	Later bedtime associated with myopia onset. No association between sleep duration and myopia.
5.	Qu et al., 2020 [30]	Correlation of myopia with physical exercise and sleep habits among suburban adolescent	Retrospective cross-sectional study Qungpu/China	1,831	11 to 18 years	Questionnaire	Self-report	Sleep habits like late bedtime and wake time are associated with myopia. No significant association with sleep duration.
6.	Wei et al., 2020 [35]	Sleep duration, bedtime, and myopia progression in a 4-year follow-up of Chinese children: the Anyang Childhood Eye Study	4-year school-based follow-up Beijing/China	1,887	7.09+/-0.41	Questionnaire	Cycloplegic refraction	Myopia progression and axial elongation inversely related to sleep duration in girls.
7.	Qi et al., 2019 [32]	Risk factors for incident myopia among teenaged students of the experimental class of the Air Force in China	3-year prospective longitudinal cohort study/China	522 boys	14-16 years	Questionnaire	Cycloplegic refraction at baseline and follow-up	Sleep duration of less than 49 hours per week not a significant risk.
8.	Huang et al., 2019 [33]	The prevalence of myopia and the factors associated with it among university students in Nanjing	Cross-sectional among the university students in Nanjing/China	1,153	19.6+/-0.9 years	Self-reported questionnaire	Self-reported use of glasses or contact lens	Sleep duration myopia has no association.
9.	Sensaki et al., 2018 [34]	Sleep duration in infants was not associated with myopia at 3 years	Birth cohort/Singapore	376	Infants	A brief infant sleep questionnaire filled by the caregiver at 12 months of age	Cycloplegic autorefraction	Sleep duration and quality at 12 months of age were not associated with refractive error at three years.
10.	Zhou et al., 2017 [27]	Association between parents’ attitudes and behaviors toward children’s visual care and myopia risk in school-aged children	Survey Wuhan/China	894	11.37±2.83.	Questionnaire filled by parents	Non-cycloplegic and later cycloplegic for those found myopic	Children whose parents ensured for their sufficient sleep had a decreased myopia.
11.	Ayaki et al., 2016 [19]	Decreased sleep quality in high myopia children	Cross-sectional survey/Japan	486	10-59 years (10-19 years -278)	The Pittsburgh Sleep Quality Index (PSQI)	Non-cycloplegic refraction using autorefractor	Myopic children slept one hour less than non-myopes. High myopia in children is associated with shorter sleep duration.
12.	Jee et al., 2016 [24]	Inverse relationship between sleep duration and myopia	Cross-sectional study/Korea	3,625	12-19 years	Standardized interview	Autorefraction without cycloplegia	Risk for myopia was decreased in those with more than 9 hours of sleep.
13.	Zhou et al., 2015 [31]	Disordered sleep and myopia risk among Chinese children	Report as part of clinical trial Guangzhou/China	1970	9.8 years +/-0.44	Parents answered children's sleep habit questionnaire	Automated cycloplegic refraction	No significant association between sleep duration and myopia.
14.	Gong et al., 2014 [25]	Parental myopia, near work, hours of sleep, and myopia in Chinese children	Cross-sectional Survey/ China	15316	6 to 18 years	Interview using a predesigned questionnaire	Non-cycloplegic refraction	Children who slept for less than 7 hours were more likely to have myopia.
15.	Li et al., 2022 [36]	Sleep patterns and myopia among school- aged children in Singapore	Cross-sectional survey/ Singapore	572	9 years at the analysis	Children’s Sleep Habits Questionnaire (CSHQ)	Cycloplegic refraction	Sleep duration was not independently associated with myopia.
16.	Huang et al., 2023 [37]	Association between sleep duration and myopia among Chinese children during the COVID-19 pandemic	Cross-sectional survey/China	1140	6 to 18 years	Standardized questionnaire	Cycloplegic refraction	Sleep duration was related to myopia, cycloplegic SE, and AL among Chinese children during the COVID-19 pandemic-related lifestyles, but no independent association.

Discussion

Many studies have confirmed the role of healthy sleep on normal human development; however, sleep deprivation is pervasive and affects various aspects of health, including vision. Inspired by the animal and epidemiological studies suggesting the role of sleep in refraction development, we felt it is essential to critically review the role of sleep duration in the development and progression of myopia.

It is known that during development, the axial length of the eye increases to match the optical power, and thus light rays are focused on the retina. Any influences that cause deviation from this homeostatic process of eye growth may result in myopia. The rising prevalence of myopia worldwide has led to the search for factors other than genetics that might have a role in the development and progression of myopia. These factors include excessive near work, higher education, economic status, and less time spent outdoors. Recently, cross-sectional [38,39], longitudinal [40], and even interventional studies [41] have suggested that outdoor time in sunlight has a preventive influence on myopia development. Laboratory animal studies show that increased outdoor sunlight exposure reduces eye growth by increasing dopamine release in the retina [42,43].

In addition, the influence of circadian light-dark pattern/sleep-wake cycles to modulate refractive development in the eye is observed in myopia animal models, like a chick, mouse, and *Drosophila melanogaster* [13,44]. When the light stimulates retinal photoreceptors, melatonin synthesis is inhibited, and dopamine is released [18]. These two mutually inhibitory hormones are known to coordinate the circadian system. Thus, dysregulation influenced by environmental light exposure patterns may also disturb the homeostatic balance in the growth of the visual system [45].

Furthermore, myopes have higher melatonin levels compared to emmetropes, emphasizing the role of intact circadian rhythms, like regular sleep for normal eye development [46]. Some also suggest that the inactivity of the ciliary muscle during sleep could prevent or alleviate myopic progression [25,47]. Thus, there is considerable overlap between biological pathways controlling sleep and ocular development.

This comprehensive review of the literature suggests that apart from other environmental factors, an individual's sleep has a role in developing myopia. Although only six studies found a significantly shorter sleep duration in myopes, all the studies that have studied this relationship show a trend of association between the two. One of the reasons for finding few studies showing significant association might be that there is only recent interest in the role of sleep, and earlier studies that had explored various environmental and behavioral factors did not objectively record sleep duration in association with myopia. This points to the fact there was less awareness of the role of sleep in refraction development.

The limitation of this review is the paucity of research on the possibility of an association between sleep duration and myopia. This lack of evidence could be explained by the relatively recent interest in learning more about how sleep affects the development of myopia. Prior studies did not explicitly measure the duration of sleep in connection to myopia; instead, they concentrated mostly on a range of behavioral and environmental factors, as well as the lack of consistency and standardization among studies. Variations in methodology pertaining to the duration of sleep and myopia assessments, with irregularities in participant demographics, complicate the interpretation of findings.

Although this systematic review increases our understanding of the link between sleep duration and myopia, it is crucial to recognize and tackle these limitations by adopting uniform procedures to evaluate refractive error and sleep factors which may improve the consistency and strength of results from various research.

A study of nine-year-olds from diverse backgrounds found no direct link between sleep patterns and myopia, suggesting that other factors, like education and outdoor time, are more important. Sleep patterns may just reflect these influences, such as increased screen time or near-work activities. Further research is needed to clarify if activities, like near-work before bedtime, affect sleep quality or if they directly impact myopia risk [36,37]. Similarly, a study of 1,140 Chinese children aged six to 18 during the COVID-19 pandemic found sleep duration correlated with myopia, cycloplegic spherical equivalent (SE), and axial length (AL), but no independent link was identified. The studies that failed to demonstrate an association between the two still reported that myopes were sleeping for fewer hours than non-myopes. Some authors have even said that those who had shorter sleep duration had high myopia. This information is significant as sleep is an essential modifiable behavioral factor.

A preliminary look at the studies showed that the majority of these studies are done in Asia. However, this is not a regional problem as analysis of prevalence data shows that myopia in East Asia is already high.

The significant parameter of sleep other than sleep duration is bedtime. Few studies have shown an association with bedtime. Some authors have even suggested that it is not the duration of sleep but bedtime and rise time that is significant in the development of visual acuity. Ayaki et al. found that myopes had a later bedtime than emmetropes, and the latest bedtime was noted in high myopes than low myopes and no myopes [19].

The circulating melatonin was significantly higher in myopes compared with non-myopes [48,49]. Sleep is fundamentally regulated by melatonin synthesis, and release and retinal dopaminergic pathways reciprocally control this circadian cycle and consequentially regulate eye growth. Therefore, disruption in the circadian rhythms can cause dysregulated ocular growth. The molecular mechanisms have shown that sleep and myopia are interrelated due to retinal dopaminergic pathways. Melatonin is the principal chemical mediator of sleep and is in a reciprocal relationship with dopaminergic retinal pathways. Thus, sleep deprivation leading to disturbances in dopaminergic pathways may result in axial elongation and myopia development [50]. 

Studies have shown that more time spent in near work and less time in outdoor activities seem to contribute to the pathogenesis of myopia; even this can also be seen as a covariate to shorter sleep duration [51].

All included studies used self-reported measurements with questionnaires filled by the parents or children to evaluate sleep time. Thus, the indirectly collected data represent a potential source of error. Despite the lack of research in this area, our review has examined the association of sleep duration, which is a changeable habit. The emphasis on the alignment of body rhythms with circadian rhythms may be a cheaper intervention and thus help decrease the economic burden due to myopia and its related complications. Longitudinal studies may improve the reliability and generalizability of findings.

## Conclusions

Although there is a lot of variability in sleep duration in the population with myopia, most have pointed toward the alarming sleep trends that can contribute to myopia development. By identifying the role of sleep duration and early bedtimes, this review may be significant.

Sleep habits are a modifiable behavior and can be promoted among the young generation. Future studies on myopes are needed to ascertain whether the sleep duration or the timing of sleep and wakefulness are associated with the onset of myopia and the progression of myopia in childhood.
